# Differences in Bioenergetic Metabolism of Obligately Alkaliphilic *Bacillaceae* Under High pH Depend on the Aeration Conditions

**DOI:** 10.3389/fmicb.2022.842785

**Published:** 2022-03-18

**Authors:** Toshitaka Goto, Shinichi Ogami, Kazuaki Yoshimume, Isao Yumoto

**Affiliations:** ^1^Bioproduction Research Institute, National Institute of Advanced Industrial Science and Technology (AIST), Sapporo, Japan; ^2^Graduate School of Agriculture, Hokkaido University, Sapporo, Japan; ^3^College of Industrial Technology, Nihon University, Narashino, Japan

**Keywords:** bioenergetic mechanism, membrane electrical potential, *Bacillaceae*, *Evansella clarkii*, Donnan effect, H^+^ capacitor, cytochrome *c*, alkaliphilic

## Abstract

Alkaliphilic *Bacillaceae* appear to produce ATP based on the H^+^-based chemiosmotic theory. However, the bulk-based chemiosmotic theory cannot explain the ATP production in alkaliphilic bacteria because the H^+^ concentration required for driving ATP synthesis through the ATPase does not occur under the alkaline conditions. Alkaliphilic bacteria produce ATP in an H^+^-diluted environment by retaining scarce H^+^ extruded by the respiratory chain on the outer surface of the membrane and increasing the potential of the H^+^ for ATP production on the outer surface of the membrane using specific mechanisms of ATP production. Under high-aeration conditions, the high ΔΨ (ca. -170 mV) of the obligate alkaliphilic *Evansella clarkii* retains H^+^ at the outer surface of the membrane and increases the intensity of the protonmotive force (Δp) per H^+^ across the membrane. One of the reasons for the production of high ΔΨ is the Donnan potential, which arises owing to the induction of impermeable negative charges in the cytoplasm. The intensity of the potential is further enhanced in the alkaliphiles compared with neutralophiles because of the higher intracellular pH (ca. pH 8.1). However, the high ΔΨ observed under high-aeration conditions decreased (∼ -140 mV) under low-aeration conditions. *E*. *clarkii* produced 2.5–6.3-fold higher membrane bound cytochrome *c* in the content of the cell extract under low-aeration conditions than under high-aeration conditions. The predominant membrane-bound cytochrome *c* in the outer surface of the membrane possesses an extra Asn-rich segment between the membrane anchor and the main body of protein. This structure may influence the formation of an H^+^-bond network that accumulates H^+^ on the outer surface of the membrane. Following accumulation of the H^+^-bond network producing cytochrome *c*, *E*. *clarkii* constructs an H^+^ capacitor to overcome the energy limitation of low aeration at high pH conditions. *E*. *clarkii* produces more ATP than other neutralophilic bacteria by enhancing the efficacy per H^+^ in ATP synthesis. In low H^+^ environments, *E*. *clarkii* utilizes H^+^ efficiently by taking advantage of its high ΔΨ under high-aeration conditions, whereas under low-aeration conditions *E*. *clarkii* uses cytochrome *c* bound on its outer surface of the membrane as an H^+^ capacitor.

## Introduction

Extremophiles can thrive in extreme environments such as low or high temperatures, high or low pH, UV radiation and high salinity (Satyanarayana et al., 2005; Rampelotto, 2013). Such environmental adaptation mechanisms enable the species to exploit environments that are disadvantageous to the survival of other organisms in general, and to protect their biological systems from harsh environments. By studying the mechanisms of environmental adaptations of such microorganisms, it is possible to obtain an understanding of the mechanisms for sustaining life systems that could not be elucidated when studying organisms in ordinary environments. In other words, by observing the biological system from a different perspective, we may be able to understand the true meaning of the mechanism of the biological system. Environmental adaptation by microorganisms is achieved by the accumulation of slight modifications in their structural components and mechanisms while sharing basic life form principles with many other organisms. The factors related to environmental adaptation are related to each other. Therefore, a comprehensive perspective is necessary to understand the mechanism underlying environmental adaptation of microorganisms.

Reduction in intracellular pH and suppression of the pH difference between intracellular and extracellular spaces of less than 2 pH units is necessary for survival and adaptation of *Bacillaceae* in an alkaline environment. In addition, establishing a system that reduces the utilization of H^+^ in various solute transportation systems will also be important for adapting to high pH. If complete ion transportation occurs using Na^+^, it would not be necessary to enhance the efficiency of H^+^ utilization. However, alkaliphilic *Bacillaceae* strains are thought to utilize both H^+^ (e.g., ATP synthase) and Na^+^ potentials (e.g., solute transport) for survival under H^+^-deficient conditions (Dimroth and Cook, 2004). This is probably due to certain advantages of employing the respiratory chain system using H^+^. While solute transportation and flagellar rotation are performed by Na^+^/solute symporters, voltage-gated Na^+^ channels (NavBP), and a Na^+^-dependent flagellar motor stator (MotPS), the ATP production is performed by the H^+^-based ATPase (Kitada et al., 1982; Ito et al., 2004a,b; Padan et al., 2005). The Na^+^/H^+^ antiporter, Mrp (Sha), plays an important role in sustaining the Na^+^ cycle system in the cells of both *Alkalihalobacillus halodurans* C-125 and *Alkalihalobacillus pseudofirmus* OF4 (Hamamoto et al., 1994; Krulwich et al., 2001). The Na^+^ efflux conducted by this antiporter is enhanced by activation of the respiratory chain in *A*. *halodurans* C-125, indicating that the antiporter plays a role in translating the H^+^-based transmembrane potential (PMF) produced by the respiratory chain to the Na^+^-based potential (SMF), which is concomitant with the necessity to increase the intracellular H^+^ concentration. The protonmotive force (Δp) generated by the respiratory chain sustains not only ATP production, which is driven by the ATPase but also the Na^+^ cycle system, which regulates cell homeostasis in *A*. *halodurans* C-125. To sustain the combination of energy production and solute transportation systems, H^+^ and Na^+^ should be localized in the vicinity of the membrane, and a large background membrane potential (ΔΨ) is necessary. It can be presumed that H^+^ localizes to the respiratory system and the SMF is the predominant potential across the membrane.

Although there are certain variations depending on the species or strains, alkaliphilic *Bacillaceae* strains have acidic secondary cell walls. For example, *A*. *halodurans* C-125 possesses an acidic secondary cell wall consisting of teichuronopeptide and teichuronic acid (Aono and Horikoshi, 1983; Aono et al., 1993, 1995, 1999). These acidic components accompanied by high negative charge will attract H^+^ around the cell surface and consequently delay the rapid loss of H^+^ from the cell surface to the bulk high pH. In addition, the formation of the negatively charged secondary cell wall results in a lower pH inside of the cell wall than the extracellular pH (Tsujii, 2002). Slower growth was observed in a mutant deficient in the cell surface layer (S-layer) protein A (SlaA), especially when the Na^+^ concentration was low in the medium of *A*. *pseudofirmus* OF4 (Gilmore et al., 2000). Similar to other secondary cell wall components in alkaliphilic *Bacillaceae* strains, SlpA is also an acidic component (p*I* = 4.36). It was considered that the abundance of SlpA attracts H^+^ and expels OH^–^ on the cell surface. Thus, alkaliphilic *Bacillaceae* strains possess negatively charged cell wall components that protect intracellular metabolic activities by preventing equilibration with the harsh extracellular alkaline phase.

Alkaliphilic *Bacillaceae* strains mitigate alkaline environments by producing acid to avoid direct interactions with harsh environments. Many alkaliphilic *Bacillus* species have been reported to produce acid to reduce the ambient pH (Horikoshi, 2006; Hirota et al., 2013). Although acid production by bacteria is often a byproduct of carbohydrate metabolism, acid production by alkaliphilic *Bacillaceae* strains can often be observed even in media lacking carbohydrates. In such cases, acid may be produced by the deamination of amino acids by amino acid deaminases (Mano, 2020). Acid production may influence to the reduction of intracellular pH, as well as increase the availability of H^+^ in the vicinity of the cell surface and outer surface of the membrane. In addition, cells produce acids as a group, and it can be presumed that acid production also alleviates the surrounding alkaline environments. Thus, acid production by alkaliphiles is considered an important factor for adaptation to high pH.

As described above, alkaliphilic bacteria comprise exquisite total adaptation mechanisms to mitigate harsh environments, which is one-thousandth of the normal H^+^ concentration compared to neutral pH. However, ATP production through mechanisms that require a transmembrane pH gradient (outside < inside) is still difficult. In this review, we aimed to understand how alkaliphilic bacteria synthesize ATP, in alkaline environments by comparing the bioenergetic parameters in an obligate alkaliphilic bacterium (cannot grow in neutral pH), *Evansella clarkii*, a facultative alkaliphilic bacterium (can grow in both alkaline and neutral pH), *Sutcliffiella cohnii*, and a neutralophilic bacteria (can grow only in neutral pH), *Bacillus subtilis*, by changing the aeration conditions. Oxygen is the final electron acceptor in the aerobic respiratory chain. Therefore, the limitation of oxygen will present an additional stress factor to the bacteria. By comparing the parameters related to energy production metabolism in alkaliphilic bacteria, we can understand the regulation of bioenergetic factors. Based on this observation, we will be able to understand the relationship between bioenergetic factors and adaptation mechanisms in the corresponding environment.

## Taxonomic Background of Alkaliphilic *Bacillaceae*

**FIGURE 1:**
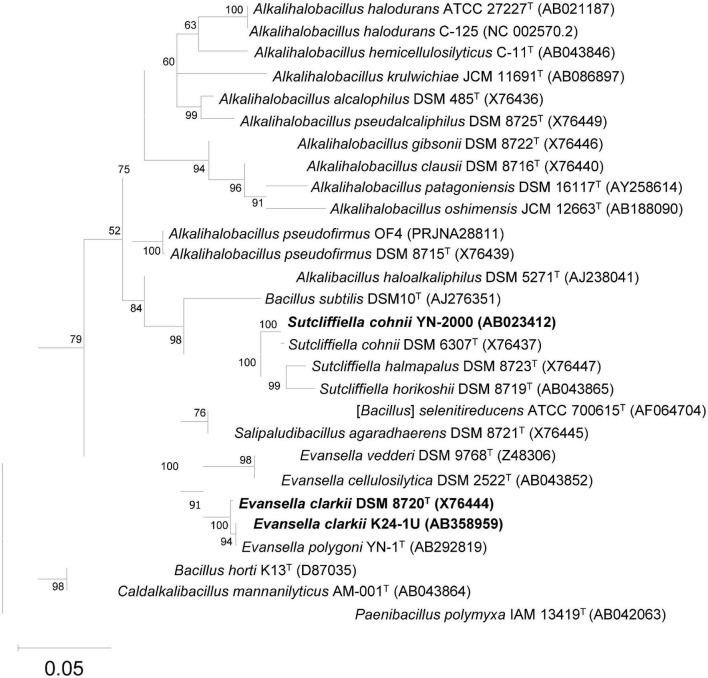
Maximum-likelihood phylogenetic tree derived from 16S rRNA gene sequences of obligately alkaliphilic *Evansella clarkii* and other related alkaliphilic and neutralophilic *Bacillaceae*. General time reversible model (Nei and Kumar, 2000) was used as the evolutionary model. The tree with the highest log likelihood (-7837.68) is shown. Initial tree(s) for the heuristic search were obtained automatically by applying Neighbor-Join and BioNJ algorithms to a matrix of pairwise distances estimated using the maximum composite likelihood (MCL) approach, and then selecting the topology with a superior log likelihood value. A discrete gamma distribution was used to model evolutionary rate differences among sites [5 categories (+ G, parameter = 0.4765)]. The rate variation model allowed for some sites to be evolutionarily invariable [(+ I), 55.96% sites]. Phylogenetic positions of the obligately alkaliphilic *E*. *clarkii* DSM 8720*^T^* and K24-1U, and the facultatively alkaliphilic *Sutcliffiella cohnii* YN-2000 are indicated in bold. The bootstrap values (> 50%) based on 1000 replications are shown at the branch node. *Paenibacillus polymyxa* IAM 13419*^T^* was used as an outgroup. Bar, 0.05 substitution per nucleotide position. Evolutionary analyses were conducted in MEGA11 (Tamura et al., 2021).

Alkaliphiles are defined as microorganisms, mostly bacteria, that exhibit higher growth intensity at pH ≥ 9. Since Vedder (1934) first isolated alkaliphilic *Bacillaceae*, *Bacillus alcalophilus* (*Alkalihalobacillus alcalophilus*), many alkaliphilic *Bacillaceae* strains have been isolated from various common environments such as garden soil, feces, and horse manure. Although it is strange that microorganisms adaptable to alkali conditions are isolated from mundane environments, this may indicate that it is not uncommon for ammonia production from plant or animal decays to temporarily flow into common environments. For example, following indigo fermentation originating from composted Chinese or Japanese indigo [*Persicaria tinctoria* (Aiton) Spach] used in China, Korea, and Japan, many alkaliphilic *Bacillaceae* species such as *Sutcliffiella* spp. (including *Sutcliffiella cohnii*), *Evansella* spp. (*including Evansella polygoni*), and *Amphibacillus* spp. have been isolated and detected using 16S rRNA gene-based next-generation sequence analyses (Lopes et al., 2021a,b; Tu et al., 2021). Another possible reason for the wide distribution of alkaliphiles in common environments is the existence of micro-alkaline environments, for example, in the intestines of termites. Using 16S rRNA gene clones derived from the contents of the intestine of termites [*Termes comis* (*Termitinae*)], many alkaliphilic *Bacillaceae* species, such as those belonging to *Sutcliffiella* spp. and *Alkalihalobacillus* spp., have been detected (Thongaram et al., 2003). Although many alkaliphilic *Bacillaceae* strains had been isolated until the mid-1990s, it was unclear whether alkaliphilic *Bacillaceae* involved numerous species because of the absence of studies based on their 16S rRNA gene sequences with reference to a gene sequence database. Since the determination of the 16S rRNA gene sequence and its application in bacterial taxonomic studies, the taxonomic position of alkaliphilic *Bacillaceae* has been elucidated, and the existence of many authorized species has been confirmed (Nielsen et al., 1994, 1995; Nogi et al., 2005). Although many identified alkaliphilic species were originally considered to belong to the genus *Bacillus*, their reclassification into several new genera has been proposed following the development of genomic analysis (Gupta et al., 2020; Patel and Gupta, 2020). The following strains were used for alkaline adaptation physiological studies were as follows: *Bacillus halodurans* C-125, *Bacillus alcalophilus*, *Bacillus firmus* RAB, and *Bacillus pseudofirmus* OF4, which were reclassified as the genus *Alkalihalobacillus*; *Bacillus cohnii* YN-2000, which was reclassified as the genus *Sutcliffiella*; and *Bacillus clarkii* DSM 8720*^T^* and K24-1U, and *Bacillus polygoni*, which were reclassified as the genus *Evansella* (Figure 1).

## Membrane Lipids

Membrane lipid characteristics play an important role in conferring the bioenergetic features of bacteria owing to the respiratory components associated with the membrane. In addition, membrane lipids consistently define H^+^ permeability across the membrane, and the head group of phospholipids influences the membrane surface charges. Furthermore, when the respiratory chain exhibits the function of an H^+^ capacitor, it is possible that the H^+^ retaining capacity may change depending on the properties of the membrane lipids. Clejan et al. (1986) performed a comparative study on the composition of total membrane lipids, neutral lipids, and polar lipids, in addition to fatty acids composition, in membrane lipids using neutralophilic *Bacillus subtilis*, two strains of obligate alkaliphiles (*Alkalihalobacillus pseudofirmus* RAB and *A. alcalophilus* ATCC 27647*^T^*), and two strains of facultative alkaliphiles (*Alkalihalobacillus* sp. OF1 and *Alkalihalobacillus pseudofirmus* OF4). The total membrane lipids of obligate alkaliphiles are approximately 1.5-fold higher than those of neutralophilic *B*. *subtilis*, while those of facultative alkaliphiles are 1.1–1.4 times higher than those of neutralophilic *B*. *subtilis*. The ratio of neutral/polar lipids was higher in the two obligate alkaliphiles (approximately 82%) than that in *B*. *subtilis* (approximately 43%) and facultative alkaliphiles (33–54%). In facultative alkaliphiles the neutral lipid ratio was higher in cells grown at pH 7.5 than in those grown at pH 10.5. These results suggest that obligate alkaliphiles are fundamentally different from facultative alkaliphiles in their environmental adaptation strategies. All alkaliphiles contain appreciable amounts of squalene and C_40_ isoprenoids, whereas *B*. *subtilis* does not harbor them. Although the roles of squalene and C_40_ isoprenoids have not yet been clarified, these neutral lipids may play an important role in alkaline adaptation to alkaline environments. Among polar lipids phosphatidylglycerol (PG) content was higher in *B*. *subtilis* than in obligate and facultative alkaliphiles. Cardiolipin (CL) content was higher in obligate and facultative alkaliphiles than in *B*. *subtilis*. In facultative alkaliphiles, CL content was not always higher in the cells grown under an alkaline pH than in those grown under a neutral pH medium. Enomoto and Koyama (1999) examined polar lipid content using facultative alkaliphiles *Sutcliffiella cohnii* YN-2000 and *Exiguobacterium aurantiacum* BL77/1 grown at pH 7.5 and pH 10 each. The results showed that CL content was higher in cells grown at pH 10 in both the strains. Both CL and PG contents increased in the cells grown at pH 10 in strain BL77/1. They also estimated the negative ion capacity of the membrane and found that the negative surface charge was higher in the cells grown at pH 10 than in cells grown at pH 7.2 in both strains. Therefore, it was concluded that the increase in the negative surface charge of the cells grown at pH 10 was attributed to the increase in acidic phospholipids CL and PG. Clejan et al. (1986) reported that branched-chain fatty acids and unsaturated fatty acids are higher in obligate alkaliphiles than in facultative alkaliphiles. However, it has been reported that facultative alkaliphilic *S*. *cohnii* strains (YN-2000 and DSM 6307*^T^*) exhibit much higher unsaturated fatty acid contents than obligate alkaliphilic *A*. *alcalophilus* JCM 5262*^T^*. Unsaturated fatty acid content was lower in the cells grown at pH 10 than in those grown at pH 7 (Yumoto et al., 2000). The obligate alkaliphilic *Evansella polygoni* YN-1*^T^* contains 22.1% unsaturated fatty acid within total fatty acids, whereas *Evansella clarkii* DSM 8720*^T^* does not contain any unsaturated fatty acid (Aino et al., 2008). However, *E*. *clarkii* DSM 8720*^T^* contains 92.5% branched chain fatty acids within total fatty acids. Since membrane lipids will change depending on the culture conditions, it is difficult to compare the result from different reports. The reported facts described above suggest that the strategies for alkaline adaptations of *Bacillaceae* differ depending on the strain. Therefore, it is important to understand the role of membrane lipids during environmental adaptation to determine the interrelationships between other related factors.

## Vertically Localized Bioenergetic Parameters in Membrane Surface

It has been considered that the protonmotive force (Δp), which drives F_1_F_0_-ATPase, consists of the following components: ΔpH (higher extracellular H^+^) and the ΔΨ (larger intracellular negative charge) across the membrane (Mitchell, 1961).


Δ⁢p=Δ⁢Ψ-Z⁢Δ⁢pH

$$Z=2.3\,\mathrm{RT}/F=\mathrm{ca}\,.59\,\mathrm{mV}\,\left(\mathrm{at}\,25^{{}^{\circ}}\,C\right),$$

where R = gas constant (8.315 J⋅K^–1^⋅mol^–1^), T = absolute temperature (298 K = 25°C), and F = Faraday constant (96.485 kJ⋅mol^–1^⋅V^–1^).

In general, the bioenergetic parameters, ΔΨ and ΔpH, estimated in the bulk base are considered for application to the formula described above. One of the reasons for this is the difficulty in measuring the real ΔpH and ΔΨ in the vicinity of the outer surface of the membrane at the entrance of the F_1_F_0_-ATPase. However, in the case of alkaliphilic *Bacillaceae* strains, there are several specific factors affecting the membrane surface ΔΨ and ΔpH: (1) the estimated ΔΨ cannot account for the intensity of each H^+^ for ATP production, (2) modified proteins located on the outer membrane surface may facilitate the transfer of H^+^ on the outer surface of the membrane, and (3) the presence of phospholipids having negatively charged head groups on the outer surface of the membrane may create a localized low pH microlayer at the site.

Bioenergetic parameters and growth features have been estimated in facultative alkaliphilic *A*. *pseudofirmus* OF4 under pH-controlled culture conditions (Sturr et al., 1994). Strain OF4 exhibited a specific growth rate and measurable Δp (bulk-based) of 1.10 h^–1^ and -26 mV, respectively, at pH 10.6. On the other hand, the strain exhibited a specific growth rate and Δp of 0.77 h^–1^ and -140 mV, respectively at pH 7.5. Thus, the faster growth rate in pH 10.6 than that in pH 7.5 cannot account for using the measurable bioenergetic parameters. This discrepancy between the growth features and bioenergetic parameters can be observed in other alkaliphilic *Bacillaceae* strains. The facts described above indicate that bulk-based or measurable bioenergetic parameters cannot account for the growth of alkaliphilic *Bacillaceae* strains.

Although the contribution of the phospholipid head group to the accumulation of H^+^ is expected to be high, we do not presently have data corresponding to obligate alkaliphilic *Evansella clarkii*. However, we found specific segments that may contribute to the H-bond network associated with the structure of membrane-bound cytochrome *c*. Here, enhanced efficiency in H^+^ usage by reducing H^+^ diffusion and the increasing H^+^ concentration available for the ATP production in *E. clarkii* are discussed below.

## Characteristics of Cytochrome *C* Expression Following Bacterial Adaption to Different PH Values

It is known that cytochrome *c* is released from mitochondria when cells are stimulated to induce apoptosis. Thus, it is known that the function of cytochrome *c* is multifaceted. The primary role of cytochrome *c* as a component of the respiratory chain is to transfer electrons from the cytochrome *bc*_1_ complex (complex III) to the cytochrome *c* oxidase (complex IV). Certain alkaliphilic bacteria, such as *S*. *chonii* and *E*. *clarkii*, are known to exhibit higher cytochrome *c* contents than neutralophilic *Bacillaceae*, such as *B*. *subtilis*. However, the specific functions related to the alkaline adaptation have not yet been clarified. To understand the molecular features of the intact protein, it is indispensable to consider the function of cytochrome *c*. Some of the cytochromes *c* from *Bacillaceae* strains have been reported to be in soluble forms due to the lacking of an N-terminal sequence associated with intrinsic protease activity (Yamaguchi et al., 1966; Miki and Okunuki, 1969a,b; Woolley, 1987; Davidson et al., 1988; Benini et al., 1998, 2000). Although the purification of a protein using a cocktail of protease inhibitors is promising, the inhibitors do not always inhibit protease activity adequately. Therefore, we attempted to purify intact membrane-bound cytochrome *c* from obligate alkaliphilic *E*. *clarkii*, which exhibited weak protease activity. Here, the molecular features of membrane-bound cytochromes *c* from neutralophilic *B*. *subtilis*, and facultative and obligate alkaliphiles are described. Comparison of cytochromes *c* derived from three categories of *Bacillaceae* (neutralophiles, facultative, and obligate alkaliphiles) revealed the peculiarities of cytochromes *c* obtained from obligate alkaliphiles.

### Cytochrome *c* in Neutralophilic *Bacillus subtilis*

**FIGURE 2:**
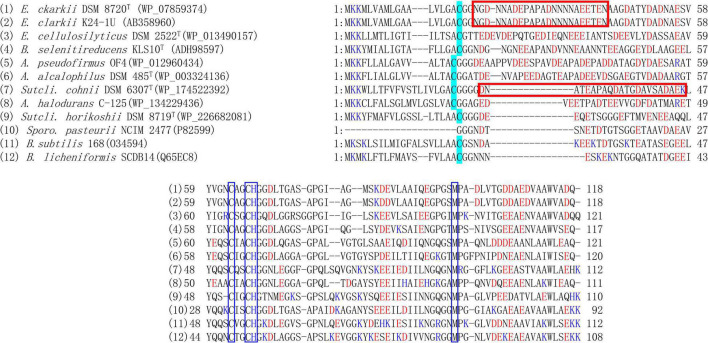
Amino acid sequence alignment of membrane bound cytochrome *c*-550 from *Evansella clarkii* and other alkaliphilic and neutralophilic *Bacillaceae*. Asn (N)-rich segment, Asn (N)^21^-Asn (N)^43^ [Asn (N)^4^-Asn (N)^26^ in the processed protein base] in the *E. clarkii* cytochromes *c* is indicated by red box. The acid residue-abundant segment of Glu^25^-Lys^46^ (Glu^6^-Lys^27^ in the processed protein base) in *S. cohnii* DSM 6307^T^ is indicated by red box. N-terminal amino acid residue in processed cytochrome *c*, [Cys (C)] is indicated by blue marker. The blue boxed sequences are heme sequences representing heme-binding site and axical ligands (H and M). Acidic and basic residues are indicated by blue and red letters. Although *A*. *pseudofirmus* OF4 is a facultative alkaliphile, the species *A*. *pseudofirmus* and the presented cytochrome *c* sequence are classified in obligate alkaliphile. Although *Sporosarcina pasteurii* is an obligate alkaliphile, its cytochrome *c* sequence is similar to those in the facultative alkaliphilic strains.

**FIGURE 3:**
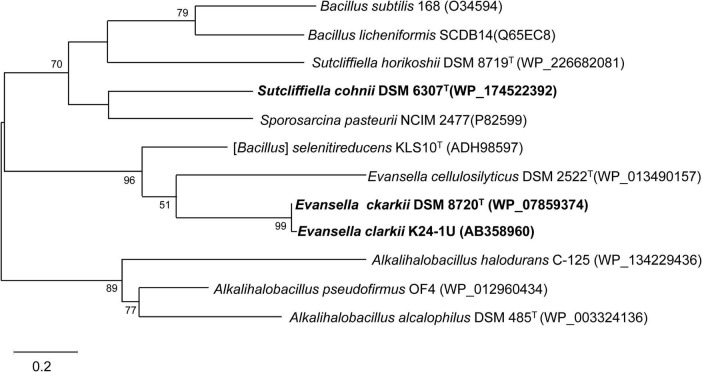
Maximum-likelihood phylogenetic tree of membrane bound cytochromes *c* from *Evansella clarkii* and other alkaliphilic and neutralophilic *Bacillaceae*. The evolutionary history was inferred by using the Whelan and Goldman model (Whelan and Goldman, 2001). The tree with the highest log likelihood (-2371.51) is shown. The percentage of trees in which the associated taxa clustered together is shown next to the branches. Initial tree(s) for the heuristic search were obtained automatically by applying Neighbor-Join and BioNJ algorithms to a matrix of pairwise distances estimated using the JTT model, and then selecting the topology with a superior log likelihood value. A discrete Gamma distribution was used to model evolutionary rate differences among sites [5 categories (+ *G*, parameter = 1.7094)]. Phylogenetic positions of cytochromes *c* of obligately alkaliphilic *E*. *clarkii* are indicated in bold. Bar, 0.2 substitution per nucleotide position. Evolutionary analyses were conducted in MEGA11 (Tamura et al., 2021).

Since many *Bacillaceae* strains exhibit strong protease activity, it is difficult to purify intact cytochrome *c*. Although *Bacillaceae* strains should not have soluble cytochromes *c* due to the lack of the periplasmic space in the cell, several soluble cytochromes *c* have been purified. It has been reported that *B*. *subtilis* 168 possesses two types of membrane-binding cytochromes *c*-550 and *c*-551. Cytochrome *c*-551 has a molecular mass of 10 kDa and is composed of 92 amino acid residues, including 14 basic amino acid residues in its processed form, and binds to the membrane *via* a diacyl-glyceryl-cysteine moiety by modification of its N-terminal sequence (Figure 2 and Table 1; Bengtsson et al., 1999). Cytochrome *c*-551 is the counterpart of cytochrome *c* to cytochrome *c*-550 in obligate alkaliphilic *E*. *clarkii*. The midpoint redox potential was considered to be > + 100 mV and its p*I* was 3.8. However, the function of cytochrome *c*-551 has not yet been elucidated. It is speculated that cytochrome *c*-551 is involved in the electron transfer between cytochrome *bc*_1_ and cytochrome *c* oxidase. The phylogenetic position of cytochrome *c*-551 was similar to that of *Bacillus licheniformis* SCDB cytochrome *c* (Figure 3). Cytochrome *c*-550 has a molecular mass of 13 kDa and is composed of 120 amino acid residues with a membrane anchor domain consisting of a single α-helical transmembrane segment of a hydrophobic polypeptide comprising 30 amino acid residues and a heme- containing main body protein comprising approximately 74 residues with a calculated p*I* of 5.4 (von Wachenfeldt and Hederstedt, 1990). The midpoint redox potential of cytochrome *c*-550 is + 178 mV (von Wachenfeldt and Hederstedt, 1993). The function of cytochrome *c*-550 is electron transfer from cytochrome *b*_6_*c* to the cytochrome *caa*_3_ terminal oxidase, and the main supercomplex formed by *caa*_3_ oxidase, the *b*_6_*c* complex, and cytochrome *c*-550 with ATP synthase has been isolated (García Montes de Oca et al., 2012).

**TABLE 1:** Biochemical properties of membrane bound cytochromes *c* of obligate alkaliphilic *Evansella clarkii*, facultative alkaliphilic *Sutcliffiella cohnii*, and neutralophilic *Bacillus subtilis*, and *Geobacillus* PS3.

	*Evansella clarkii* cytochrome *c*-550^a^	*Sutcliffiella cohnii* cytochrome *c*-553^b^	*Bacillus subtilis* cytochrome *c*-551^c^	*Geobacillus* PS3 cytochrome *c*-551^d^
Number of amino acid residues^e^	101	93	92	93
Molecular mass in SDS-PAGE (kDa)	20, 17 (11.1)^I^	10.5 (9.7)	10 (11.0)	10.4 (10.4)
Molecular mass in gel-filtration (kDa)^f^	40 (44.4)^J^	37 (38.8)	ND^g^	33 (31.2)
Number of peak in analysis in Reverse-phase chromatography	3	ND	ND	1
Presence of Asn^4^-Asn^26^ sequence^e^	Yes	No	No	No
Acidic and basic amino acid residues between 6th and 26th residues^e^	Acidic 6 or 7; basic 0	Acidic 6; basic 1	Acidic 8; basic 3	Acidic 5; basic 1
Number of basic residues^e^	2	7	14	12
Midpoint redox potential (mV)^h^	83	87	>100	225
p*I*	4.1	3.9	3.8	4.0
Absorption maxima at				
Oxidized form (nm)	408	411	409	409
Reduced form (nm)	415, 521, 550	417, 524, 553	416, 522, 551	416, 522, 551

*^a^The data obtained from Ogami et al. (2009) using E. clarkii K24-1U and whole genome sequence of E. clarkii DSM 8720^T^ (NZ_MTIV00000000.1) were considered.*

*^b^The data obtained from Yumoto et al. (1991) using S. cohnii YN-2000 and whole genome sequence of S. cohnii DSM 8720^T^ (NZ_CP018866.1) were considered.*

*^c^The data obtained from Bengtsson et al. (1999).*

*^d^The data obtained from Sone et al. (1989) and Fujiwara et al. (1993).*

*^e^Processed proteins were considered*

*^f^Values were subtracted from the molecular mass of Triton X-100.*

*^g^ND: No data.*

*^h^Values are estimated using redox titration.*

*^I,J^Theoretical value including of heme, anchor and N-terminal modification.*

### Cytochromes *c* in Facultative Alkaliphilic *Sutcliffiella cohnii* YN-2000 and *Sporosarcina pasteurii* NCIM 2477

The abundance of membrane-bound cytochrome *c* was higher at pH 10 than that at pH 7 in facultative alkaliphilic *S*. *cohnii* YN-2000 (Yumoto et al., 1991). The elution profile of anion exchange chromatography loading of the solubilized membrane fraction by the detergent, Triton X-100 exhibited a larger amount of cytochrome *c*-553 in cells grown at pH 10 than in cells grown at pH 7 (Yumoto et al., 1991). Cytochrome *c*-553 has a molecular mass of 10.5 kDa by sodium dodecyl sulfate-polyacrylamide gel electrophoresis (SDS-PAGE) with a p*I* of 3.9 (Table 1). If cytochrome *c*-553 of strain YN-2000 is the same as that in *S*. *cohnii* DSM 6307*^T^*, it consists of 93 amino acid residues including seven basic amino acid residues in the processed protein. The midpoint redox potential was + 87 mV in the pH range of 6–8. The native molecular mass, as determined by gel filtration, was 37 kDa. Therefore, it was suggested that cytochrome *c*-553 forms a tetramer in its native form in solution or in its original membrane-binding form.

The phylogenetic position of *S*. *cohnii* DSM 6307*^T^* cytochrome *c* clustered with that of *Sporosarcina pasteurii* NCIM 2477 is shown in Figure 3. Cytochrome *c* oxidase in *S*. *cohnii* YN-2000, cytochrome *aco*_3_, which is a different type from other *Bacillaceae* cytochrome *c* oxidases was purified and characterized (Qureshi et al., 1990). Cytochrome *aco*_3_ reacts with cytochrome *c*-553, and the terminal enzymatic activity is greatly enhanced in the presence of poly-L-lysine, which accelerates interaction of two negatively charged molecules: cytochrome *c*-553 and cytochrome *aco*_3_ (Yumoto et al., 1993).

The three-dimensional (3D) structure of cytochrome *c* in alkaliphilic *Bacillaceae* was first studied in *S*. *pasteurii* cytochrome *c*-553 (Benini et al., 2000). Cytochrome *c*-553 has a molecular mass of 9.6 kDa and consists of 92 amino acids having a midpoint redox potential of + 47 mV and a predicted p*I* of 3.96 (Benini et al., 1998). Most of the charges were localized on the opposite side of that exposed to the heme edge. This localization may be related to H^+^ transfer on the outer surface of the membrane. The correlation between heme solvent accessibility and entropy suggests a direct link between the major determinant of the electrical potential (entropy) and a structural parameter (heme solvent exposure). The low midpoint redox potential of cytochrome *c* could be attributed to the decrease in reduction entropy caused by the extrusion of water molecules from the reduction of the hydration shell in this protein. This event affects solvent accessibility to the heme during its reduction. Thus, analysis of the 3D-structure revealed important structural characteristics that explain the low redox potential and retention of H^+^ at the outer surface of the membrane of the cytochrome *c* in the alkaliphile.

### Cytochromes *c* in Obligately Alkaliphilic *Alkalihalobacillus pseudofirmus* RAB and *Evansella clarkii* K24-1U

The first purified and characterized cytochrome *c* from an alkaliphilic *Bacillaceae* strain was cytochrome *c*-552 isolated from *A. pseudofirmus* RAB (Davidson et al., 1988). Cytochrome *c*-552 has a molecular mass of 16.5 kDa with an acidic p*I* of 3.4. The midpoint redox potential of this cytochrome is + 66 mV at pH 7, which decreases at pH > 8.3, depending on the pH increase. According to resonance Raman spectroscopy, this pH-dependent decrease in redox potential may be attributed to a switch in the sixth ligand of heme *c* from methionine to histidine when the oxidized cytochrome is reduced (Larsen et al., 1990). Cytochrome *c*-552 is autooxidizable and is purified in a soluble form. These characteristics may arise due to the partial digestion of cytochrome *c* by intrinsic proteinases during purification. Although most strains belong to the same species as *A. pseudofirmus* RAB are obligate alkaliphiles (Nielsen et al., 1995), whereas *A*. *pseudofirmus* OF4 is a facultative alkaliphile. If cytochrome *c*-552 in *A*. *pseudofirmus* RAB is the same as that in the corresponding cytochrome *c* in *A*. *pseudofirmus* OF4, cytochrome *c*-552 consists of 105 amino acid residues and exhibits the same branching with cytochrome *c* in obligate alkaliphilic *A*. *alcalophilus* DSM 485*^T^* (Figure 3).

The cell extract of obligate alkaliphilic *E*. *clarkii* K24-1U exhibits higher cytochrome *c* content than that in neutralophilic *B*. *subtilis* under high-aeration conditions (4.5-fold). The cytochrome *c* abundance in *E*. *clarkii* K24-1U further increases under low-aeration conditions (6.3-fold) (Hijikata, 2004; Matsuno et al., 2018). Cytochrome *c*-550 purified from *E*. *clarkii* K24-1U, exhibited very weak protease activity (Ogami et al., 2009). Characterization of cytochrome *c*-550, determination of its gene sequence, and constricted recombinant expressing C18M anchorless mutant protein enables us to understand the molecular features in native and anchorless cytochrome *c* molecules. Native cytochrome *c*-550 and C18M cytochrome *c*-550 had molecular weights of 11,083–11,115 in the analysis of the separated fractions in HPCL and 10,543 by Matrix-assisted Laser Desorption/Ionization Time-of-Flight Mass Spectrometry (MALDI-TOF-MS) with an acidic p*I* of 4.1. The midpoint redox potential was + 83 mV in the redox titration (Table 1). The purified cytochrome *c*-550 exhibited two bands of 17 kDa and 20 kDa in SDS-PAGE. The native molecular mass determined by gel filtration was 40 kDa in both native and C18M cytochromes *c*. Therefore, it is suggested that cytochrome *c*-550 forms a tetramer in its native form in solution or in its original membrane-binding form. Cytochrome *c*-550 exhibited only one band of 23 kDa in blue native-PAGE for both native and C18M cytochromes *c*-550. These numbers of SDS-PAGE and blue native-PAGE of molecular mass correspond to the dimer based on the molecular mass of the minimum unit of molecular mass determined by MALDI-TOF-MS using fractions separated by HPLC and its gene sequence. These two bands in SDS-PAGE were shifted to 10 and 15 kDa in C18M cytochrome *c*-550. Therefore, the appearance of the two bands in SDS-PAGE was not attributed to the N-terminal modifications but to the internal specific sequence located near the N-terminal sequence of Asn^21^-Asn^43^ because the specific sequence lacking *S*. *cohnii* YN-2000 tetrameric cytochrome *c* exhibited only one monomer band in SDS-PAGE (Yumoto et al., 1991). The above results suggested that the binding molecular mechanisms of dimeric and tetrameric (dimeric *plus* dimeric) forms are different.

The determined gene sequence of cytochrome *c*-550 revealed the hydrophobic N-terminal sequence Met^1^-Ala^17^ as the signal peptide sequence (Ogami et al., 2009; Figure 2). In addition, MALDI-TOF-MS analysis using fractionated by HPLC of the native protein and fatty acid analysis of cytochrome *c* revealed the structure of the modified moiety of the protein. During the processing of mature cytochrome *c* production, expressed cytochrome *c*-550 is translocated to the extracellular side of the membrane by the signal peptide. After the signal peptide was dissociated from the main body protein, terminal Cys^18^ was modified by attaching diacylglycerol and acetyl moieties. Cytochrome *c*-550 binds to the fatty acid length of C_15_ in the internal moiety and different chain lengths of fatty acids, C_15_, C_16_, and C_17_ in the external moiety *via* glycerol-Cys^18^. Molecular binding species are the major components of membrane fatty acids. Therefore, if the amount of cytochrome *c*-550 expressed fluctuates depending on the culture conditions, there will be a little influence on the fatty acid composition of the membrane.

The sulfate-reducing bacterium *Desulfovibrio gigas* possesses cytochrome *c*_3_, which has four hemes per protein molecule (Coutinho and Xavier, 1994). It has been reported that cytochrome *c*_3_ transfers H^+^ *via* a cooperative H^+^/e^–^ linkage (redox-Bohr effect) (Louro et al., 1997; Messias et al., 2006). These hemes exhibit different redox potentials by direct measurement on the electrode attached as self-assembled monolayers (SAMs) and by redox titration using mediators in a solution. This difference in redox potential in cytochrome *c* suggests changes in the redox potential depending on the distance between each heme (heme I–IV) and the electrode. Shorter distances between heme *c* and the electrode resulted in larger differences in the redox potential of the cytochrome *c* molecule between in solution status and the electrode-attached state of the cytochrome *c* molecule. These differences are attributed to the strength of the electric field (Coulomb force), which depends on the distance of each heme from the electrode (Rivas et al., 2005). The redox potential of cytochrome *c*-550 of *E*. *clarkii* measured by redox titration was + 83 mV (in a buffer solution) whereas that of cytochrome *c*-550 immobilized on the gold electrode using a SAMs of 2-amino-6-purinethiol was + 7 mV. These facts suggest that if the distances between the membrane surface and the main body cytochrome *c* differ depending on the chain length of fatty acids (C_15_, C_16_, and C_17_), the redox potential of heme *c* may change depending on their distance from the membrane surface. These electric field strength-dependent differences in redox potential may have an important role for H^+^ transfer on the outer surface of the membrane in electron transfer-coupled H^+^ transfer mechanisms.

The amino acid sequence of *E*. *clarkii* K24-1U cytochrome *c*-550 deduced from the determined gene sequence was very similar to that of cytochrome *c* in *E*. *clarkii* DSM 8720*^T^* (only one residue difference among 118 residues) (Figure 2). We collected data on bioenergetic parameters, such as O_2_ consumption and ATP production rates, in *E*. *clarkii* DSM 8720*^T^*. Therefore, we can consider these bioenergetic data combined with the data for *E*. *clarkii* K24-1U cytochrome *c*-550. Multiple alignments of two strains of *E*. *clarkii* cytochrome *c* with other cytochromes *c* from obligate and the facultative alkaliphilic and the neutralophilic *Bacillaceae* strains were constructed (Figure 2). Cytochrome *c*-550 contains much fewer basic residues (only two, including His for axial ligand of the heme *c*) compared with cytochrome *c* from facultative alkaliphiles and neutralophiles. This scarcity of basic amino acids in the obligate alkaliphilic *Bacillaceae* may be related to the acidic nature of the molecule, which expels OH^–^ and attracts H^+^ to protect the environment in the outer surface of the membrane. In the entire molecular structure, the particular part of the amino acid sequence of Asn_21_-Asn_43_ in the cytochrome *c* of the two strains of *E*. *clarkii*, which consisted of eight Asn residues and six acidic amino acid residues in 23 residues (Figure 2). The corresponding amino acid sequence of Asn_24_-Asn_37_ (Asn_7_-Asn_20_ in the processed protein base) was observed neither in the facultative alkaliphilic nor the neutralophilic *Bacillaceae* strains. This means that obligate alkaliphiles express cytochromes *c*, which express an even more effective protein for the alkaline environment adaptation than the neutralophilic or the facultatively alkaliphilic *Bacillaceae*.

Theoretically, the Asn (N) residue could influence H^+^-transfer. However, there have been few examples of the interpretation that accounts for the contribution of Asn in H^+^ transfer due to the weak hydrogen binding of this individual residue. In the catalytic reaction of methyltetrahydrofolate (MTHF) and corrinoid-iron sulfur protein (CFeSP) methyltransferase (MeTr), the methyl group of CH_3_-H_4_folate in MTHF is transferred to cob (I) amide. This reaction requires electrophilic activation of the methyl group of MTHF, which involves H^+^ transfer to the N5 group of the pterin ring of MTHF. This H^+^ transfer reaction is possible by the extended H-bond network including Asn^199^, Asp^160^, and a water molecule (Doukov et al., 2007). Thus, although Asn exhibits weak hydrogen bonds, the overall effect of this cumulative H-bond network is significant in this series reactions. Considering the cooperation of Asn and other H^+^ transferable acidic amino acid residues such as Asp or Glu, the amino acid sequence of Asn_21_-Asn_43_ in the cytochrome *c* of *E*. *clarkii* strains produces a cumulative H-bond network in the corresponding region.

Based on the reported structure of membrane-bound cytochrome *c* in *Bacillus subtilis* (David et al., 2000), the Asn-rich segment, Asn_21_-Asn_43_ (Asn_4_-Asn_26_ in the processed protein base) in *E*. *clarkii* cytochrome *c* is presumed to be located in the α-helical domain between the membrane anchor and the main body cytochrome *c* molecule. Therefore, it is considered that this region is located between the outer surface of the membrane and the main body of cytochrome *c*. Based on the tetrameric structure of this cytochrome *c* molecule, the Asn-rich segment, Asn_21_-Asn_43_, further accumulated in the corresponding location. It can be presumed that this accumulated Asn-rich segment, contributing to the intense H-bond network, influences H^+^ transfer on the outer surface of the membrane associated with the redox reaction of the main body of cytochrome *c*. The corresponding segment Asn_23_-Asn_37_ (Asn_6_-Asn_20_ in the processed protein base) was observed only in the obligate alkaliphilic *Bacillaceae* (Figure 2). If the corresponding region influences the H-bond network in the space between the main body cytochrome *c* molecule and the outer surface of the membrane, the segment of Glu_25_-Lys_46_ (Glu_6_-Lys_27_ in the processed protein base) in *S*. *cohnii* DSM 6307*^T^* may also influence the H-bond network (Figure 2). The abundance ratio of acidic to basic amino acids was higher in the facultative alkaliphilic *Bacillaceae* than in the neutralophilic *Bacillaceae* (Table 1 and Figure 2). A similar region found in membrane-bound cytochromes *c* in alkaliphilic *Bacillaceae* also contain other membrane-bound protein molecules, such as subunit II of *aco*_3_-type or *caa*_3_-type cytochrome *c* oxidase, which have abundant acidic residues and a few basic residues (Denda et al., 2001; Noor et al., 2014). This region is located in the external hydrophilic cytochrome *c*-binding domain of subunit II of the *aco*_3_-type or *caa*_3_-type cytochrome *c* oxidase. This shows that the H-bond network construction strategy, which is found in membrane-bound cytochrome *c*, is also found in the subunit structure of other protein molecules that have subunits containing cytochrome *c* segments. This configuration is probably connected to cytochrome *c*, which exhibits a low redox potential (< + 100 mV) (Orii et al., 1991). It can be predicted that the abundance of acidic amino acids, tetrameric structures, and low redox potentials are important for redox-coupled H^+^ transfer on the outer membrane surface through the H-bond network.

## Bioenergetic Parameters in Obligately Alkaliphilic *Evansella clarkii* Dsm 8720*^T^*

As described above alkaliphilic *Bacillaceae* exhibited higher cytochrome *c* content than that of neutralophilic *B*. *subtilis*. This higher content of cytochrome *c* in alkaliphiles increased further under low-aeration conditions in obligate alkaliphilic *E*. *clarkii* DSM 8720*^T^*. High- and low-aeration conditions were generated in 2 L baffled Erlenmeyer flasks with shaking at 120 rpm (rotation) and shaking at 60 rpm (rotation), respectively. This difference in cytochrome *c* content depending on the aeration condition was larger in obligate alkaliphilic *E*. *clarkii* DSM 8720*^T^* than that in facultative alkaliphilic *S*. *cohnii* YN-2000. Therefore, our aim was to understand the differences in the energy production mechanisms between high- and low-aeration conditions by estimating the H^+^ translocation rate and ATP production rate. In the present study, a comparison of the H^+^ translocation rate and ATP production rate in three categories (neutralophiles, facultative and obligate alkaliphiles) of bacteria showed the peculiarities of cytochrome *c* isolated from obligate alkaliphiles. By estimation of the intensity per H^+^ to produce ATP, we might understand the fundamental strategies of utilizing H^+^ efficiently in obligate alkaliphiles. In addition, we will understand the relationship between the molecular features of cytochrome *c* and parameters related to ATP production.

### Oxygen Consumption Rate

One of the alkaline adaptation mechanisms of alkaliphilic *Bacillaceae* is based on the enhancement of H^+^ usage efficiency. This enhanced the efficacy of H^+^ utilization in ATP synthesis and retention of H^+^ in the vicinity of the outer surface of the membrane, compared to in neutralophilic *Bacillaceae*. In fact, it has been suggested that the number of H^+^ translocated across the biological membrane from the intracellular side to the extracellular side by the respiratory chain in alkaliphilic *Bacillaceae* was significantly lower than that in neutralophilic *Bacillaceae* (Hirabayashi et al., 2012; Goto et al., 2016). The oxygen consumption rate based on the endogenous substrates of *B*. *subtilis* IAM 1026 (growth in high aeration, pH 7; O_2_ consumption was measured at pH 7) was 0.50 ± 0.06 μmol O_2_⋅min^–1^⋅mg cell protein^–1^, while that of the obligate alkaliphilic *E*. *clarkii* DSM 8720*^T^* [in high aeration, growth at pH 10; O_2_ consumption was measured at pH 10) was 0.19 ± 0.04 μmol O_2_⋅min^–1^⋅mg cell protein^–1^ (Table 2). The low oxygen consumption value is comparable to the data obtained using *A*. *pseudofirmus* OF4 (0.21 μmol O_2_⋅min^–1^⋅mg cell protein^–1^ (based on indigenous substrates in the presence of Na^+^)] (Guffanti et al., 1986). *A. alcalophilus* and *A. pseudofirmus* RAB exhibited the same O_2_ consumption rate (0.49 μmol O_2_⋅min^–1^⋅mg cell protein^–1^) when L-malate is used as a substrate (Lewis et al., 1980). Therefore, the low oxygen consumption rate of *E*. *clarkii* DSM 8720*^T^* may be attributed to the absence of the extracellular substrate. A low oxygen consumption rate compared to that of *B*. *subtilis* IAM 1026 under high aeration was also observed in facultative alkaliphilic *S*. *cohnii* YN-2000 (0.20 ± 0.07 μmol O_2_⋅min^–1^⋅mg cell protein^–1^; O_2_ consumption was measured at pH 10) under high-aeration condition. These relatively low oxygen consumption rates, compared with *B*. *subtilis* under high-aeration conditions in obligate and facultative alkaliphiles, may be attributed to the hindered translocation of the positively charged H^+^ by the respiratory chain *via* the attraction force of the intracellular negative charge due to the large ΔΨ. Thus, H^+^ transfer coupled with electron transfer in the respiratory chain may be deterred. The reduction rate of heme *a* in cytochrome *c* oxidase in vesicles reportedly increases when ΔΨ is abolished during steady-state respiration using cytochrome *c* (Gregory and Ferguson-Miller, 1989). This is probably due to the ΔΨ influencing the electron transfer from cytochrome *c* to heme *a*. Although it is expected that ΔΨ alters the redox potential of heme *a*, ΔΨ potentially inhibits the electron transfer from the outer side of the membrane (cytochrome *c*) to the inner membrane heme *a* due to repulsion between negative charges (electron negative charge vs. inner membrane negative charge by ΔΨ). Therefore, the difficulties in H^+^ transfer and electron transfer across the membrane under high ΔΨ may produce a low oxygen consumption rate in high aeration in *E*. *clarkii* DSM 8720*^T^*.

**TABLE 2:** Summary for high aeration and low aeration in obligate alkaliphilic *Evansella clarkii*, facultative alkaliphilic *Sutcliffiella cohnii*, and neutralophilic Bacillus subtilis*.

	Growth rate (μ_max_)^1^	Cytochrome *c* content (nmol ⋅ mg^–1^)^1^	Δψ (mV)^1,2^	Respiratory rate (μmol ⋅ O_2_ min^–1^ mg^–1^)^2^	H^+^/O^1^	Maximum ATP synthesis rate^2^ (nmol ⋅ min^–1^ ⋅ mg^–1^)^2^
*E. clarkii* DSM8720^T^ Low aeration	0.36	0.89 ± 0.07	−135 ± 8	ND	0.6 ± 0.1 (6)^§^	ND
*E. clarkii* DSM 8720^T^ High aeration	0.42	0.36 ± 0.01	−192 ± 3	0.19 ± 0.04	2.2 ± 0.2 (6)	26.2 ± 1.7
*S. cohnii* YN-2000 High aeration	0.38	0.62 ± 0.01	−173 ± 5	0.20 ± 0.03	2.6 ± 0.3 (6)	9.6 ± 0.9
*B. subtilis*IAM 1026 Low aeration	0.26	0.18 ± 0.06	−133 ± 13	ND	2.8 ± 0.8 (6)	ND
*B. subtilis*IAM 1026 High aeration	0.55	0.21 ± 0.02	−121 ± 7	0.50 ± 0.06	4.9 ± 0.1 (6)	2.0 ± 0.6

*These data cited from

*^1^Goto (2006) and*

*^2^Goto et al. (2016). ^§^The numbers in parentheses are based on the theoretically extruded H^+^ in 1/2O_2_ consumption by the respiratory chain.*

### H^+^/O Ratio

In the measurement of the H^+^/O ratio in alkaliphiles and neutralophiles, each measured value was obtained by culturing and measuring at pH 10 and pH 7, respectively. Since *Bacillaceae* strains do not contain complex I, which translocates 4 H^+^/O, the theoretical H^+^/O ratio in the case of *Bacillaceae* strains is thought to be 4 H^+^ from complex III plus 2 H^+^ from complex IV, equal to 6 H^+^. The H^+^/O ratio of thermophilic *Bacillaceae* (*Geobacillus*) is 6–7 (Chichen et al., 1981; Sone and Fujiwara, 1991), while those in *B*. *subtilis*, *Bacillus megaterium*, and *Bacillus licheniformis* have been reported to be approximately 4 (Jones et al., 1975). In contrast, the H^+^/O ratio in another *Bacillaceae*, *Brevibacillus brevis* was reported to be 5.01 ± 0.26 (Yaginuma et al., 1997). The variation in the H^+^/O ratio depending on the *Bacillaceae* strain may be attributed to differences in the electron flow paths in the respiratory chain.

The H^+^/O ratios under high- and low-aeration conditions in obligate alkaliphilic *E*. *clarkii* DSM 8720*^T^* were 2.2 ± 0.2 and 0.6 ± 0.1, respectively (Table 2). These values were much lower than the theoretical values. The H^+^/O ratios under high-aeration condition in facultative alkaliphilic *S*. *cohnii* YN-2000 and neutralophilic *B*. *subtilis* were 2.8 ± 0.8 and 4.9 ± 0.1, respectively (Table 2). The H^+^/O ratio in the facultative alkaliphilic strain was higher than that in the obligate alkaliphilic strain, whereas the value was lower than that in the neutralophilic strain and the theoretical values. To date, it is unclear why the H^+^/O ratios in alkaliphilic bacteria are much lower than the theoretical value. The possible reasons for these values might be due to the inhibition of translocation H^+^ by the larger ΔΨ compared with that in the neutralophiles or that the H^+^ translocated by the respiratory chain may remain on the outer surface of the membrane. However, for measuring the H^+^/O ratios, 0.15 μg/mL valinomycin and 0.85 mM KCl, which disrupt ΔΨ across the membrane, were used. Therefore, it is difficult to determine whether the reason for the low H^+^/O ratios in alkaliphiles can be attributed to ΔΨ alone.

There is a possibility that the extremely low H^+^/O ratio in obligate alkaliphilic *E. clarkii* DSM 8720*^T^* is related to the Asn-rich distinct amino acid sequence in the membrane-bound cytochrome *c*, which may influence to the H-bond network probably related to the transfer of H^+^ at the outer surface of the membrane. It is possible that this specific structure in *E. clarkii* cytochrome *c* spools up translocated H^+^ *via* the respiratory chain on the outer surface of the membrane.

The H^+^/O ratio was found to be 2.8 even under low-aeration conditions of *B*. *subtilis* (Table 2). Since the pumped H^+^ was measured in bulk in the estimation of H^+^/O, the measured values may be influenced by the activity of the respiratory chain. Thus, the H^+^/O ratio may change depending on the cell activity. In addition, there is a possibility that the expressed respiratory components may change depending on the culture conditions. Therefore, it is difficult to compare the values directly among the reported studies. Cyanide-insensitive Non-Proteinaceous Substance (NPS) with a molecular weight of 622 was found in the respiratory system of the obligate alkaliphilic *E*. *polygoni* YN-1*^T^* (Higashibata et al., 1998). Thus, it can be predicted that NPS does not influence to H^+^ translocation in the respiratory system. The terminal enzyme was a *caa*_3_-type cytochrome *c* oxidase, constituting up to only 10% of the total oxygen reducing activity, while 90% of the respiratory activity was attributed to cyanide-insensitive NPS in strain YN-1*^T^*. If the NPS is widely distributed in alkaliphilic *Bacillaceae*, it may lead to the reduction of the H^+^/O ratio. NPS may prevent the production of excess energy during the later growth phase in the effective energy production systems of alkaliphiles.

### H^+^ Translocation Frequencies Based on the Obtained Data and Theoretical Ratio

Since the O_2_ consumption rate is considered to be the turnover rate of the respiratory chain, by using the measured and theoretical H^+^/O values, the amount of H^+^ translocated by the respiratory chain when two electrons (e^–^) reduce oxygen (1/2O_2_) was estimated (2e^–^ + 2H^+^ + 1/2O_2_ → H_2_O) (Table 2). Based on the measured and theoretical H^+^/O values, translocated H^+^ by the respiratory chain is predicted to higher under low- aeration conditions than under high-aeration conditions in *E. clarkii* DSM 8720*^T^*. This prediction could be attributed to the larger ΔΨ in the high-aeration condition than that in the low-aeration condition, as described in the oxygen consumption rate section. Although the amount of translocated H^+^, based on the measured H^+^/O values, was lower in *E. clarkii* DSM 8720*^T^* [0.84 (min^–1^⋅mg protein^–1^)] than in *S*. *cohnii* YN-2000 (1.04) the translocated amounts of H^+^ using theoretical H^+^/O values were almost the same (*E. clarkii* DSM 8720*^T^*, 2.28; *S*. *cohnii* YN-2000, 2.4). The number of translocated H^+^ per 1/2O_2_ consumption in *E. clarkii* DSM 8720*^T^* is lower than that in *B*. *subtilis* IAM 1026 (6.0, high aeration) on the basis of the theoretical value. Although the above comparison used the theoretical values, it is necessary to reconsider the reality of the measured H^+^/O ratio in consideration of the actual contribution of the NPS, as described above.

If the measured values of H^+^/O reflect the actual value of the number of H^+^, it could mean that a very small number of H^+^ were translocated by the respiratory chain compared to the theoretical values. In addition, the number of translocated H^+^ in alkaliphiles was much lower than that in neutralophilic *B*. *subtilis*. The results described above are considered to indicate the physiological strategy of maintaining H^+^ on the outer surface of the membrane as much as possible and operating the cell system in an environment lacking sufficient H^+^ for ATP production and high ΔΨ as a result of the physiological environmental adaptation at high pH. Therefore, although these estimated H^+^/O values may be unrealistic, it is expected that the respiratory H^+^ translocation rate of alkaliphilic bacteria is probably lower than that of neutral bacteria.

### Efficiency of Respiratory Translocated H^+^ for ATP Production

**FIGURE 4:**
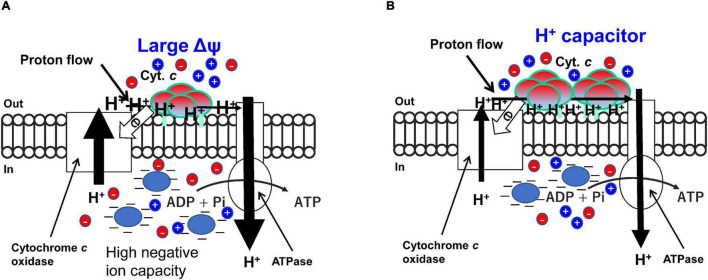
High efficiency of H^+^ located on the outer surface of the membrane based mainly on the ΔΨ and hydrogen bond network produced by the membrane bound cytochrome *c* segment in high **(A)** and low **(B)** aeration conditions, respectively in obligately alkaliphilic *Evansella clarkii*. Although the rate of respiratory translocating H^+^ is predicted to lower in high aeration **(A)**, efficiency per H^+^ for production of ATP is higher in the high-aeration conditions compared with that of in low-aeration conditions **(B)**. This difference is attributed to the larger electrical potential (Δψ) in high-aeration conditions than that in low-aeration conditions. The mechanism of the occurrence of the high Δψ may be attributed to a Donnan effect produced by membrane impermeable negatively charged substances (Blue ellipse) and heterogenous membrane permeable ions (red and blue circles). The high Δψ across the membrane may be a heavy burden for H^+^ translocation by the respiratory chain. However, the translocated protons may have a large potential for ATP production. The membrane bound cytochrome *c* content is higher in low-aeration conditions than in high-aeration conditions. The specific segment near the N-terminal sequence [Asn (N)^21^-Asn (N)^43^; Asn (N)^4^-Asn (N)^26^ in the processed protein base] in the membrane bound cytochrome *c* produces an H^+^-capacitor. The tetrameric structure and differences in the length of fatty acid anchors may have an important role for accumulation and transportation of H^+^ on the outer surface of the membrane.

To understand the effect of H^+^ translocated by the respiratory chain under high aeration conditions, the maximum ATP synthesis rates of obligate (i.e., *E. clarkii* DSM 8720*^T^*) and facultative (i.e., *S*. *cohnii* YN-2000) alkaliphiles and neutralophile (i.e., *B*. *subtilis* IAM1026) were compared on the basis of the data described in Table 2. The ATP synthesis rates of *E. clarkii* DSM 8720*^T^* were higher than those of *S*. *cohnii* YN-2000 and *B*. *subtilis* IAM 1026. This suggests that the intensity of H^+^, which is translocated by the respiratory chain to drive ATP synthesis through the ATPase, is higher than that of other bacteria, while the number of H^+^ retaining on the outer surface of the membrane is larger than in other bacteria under low aeration. This H^+^ accumulation is attributed to the H^+^-bond network producing cytochrome *c*. In addition, since *E*. *clarkii* DSM 8720*^T^* growth rate in low aeration conditions exhibits equivalent to *S*. *cohnii* in high aeration conditions and much higher than *B*. *subtilis* in low aeration conditions (Table 2), the H^+^ potential in the vicinity of the outer surface of the membrane is expected to be much higher than that measured in the bulk base. This efficient H^+^ usage may also be attributed to the H^+^ capacitor strategy on the outer surface of the membrane (Matsuno et al., 2018). The increase of cytochrome *c*-550, which possesses a specific amino acid sequence between the main body protein and membrane-anchoring part facilitates the formation of an H^+^ capacitor *via* the H-bond network. The H^+^ capacitor strategy may also influence to the maximum ATP production rate per H^+^ translocation by the respiratory chain. The predicted H^+^ utilization strategy for ATP production in high and low aeration conditions in *E. clarkii* DSM 8720*^T^* is illustrated in Figure 4. The maximum ATP production rate per H^+^ translocated by the respiratory chain of *E. clarkii* DSM 8720*^T^* under high-aeration conditions was 11.5 nmol ATP ⋅ translocated H^+–1^ (theoretical base), which is much higher than the 4.0 nmol ATP observed in *S*. *cohnii* YN-2000 and the neutralophilic *B*. *subtilis* IAM 1026 (0.33 nmol ATP ⋅ translocated H^+–1^). Thus, H^+^ efficiency under high-aeration conditions in *E. clarkii* is much higher than that in the *S*. *cohnii* and *B*. *subtilis*. This is attributed to the much higher ΔΨ in *E. clarkii* than that in the other bacteria.

## Transmembrane Electrical Potential (ΔΨ)

It has been reported that the ΔΨ in alkaliphilic *Bacillaceae* (ca. -180 to -210 mV) is larger than that in the neutralophilic *Bacillaceae* (ca. -130 mV) in the direction of ATP production (Yumoto, 2003; Goto et al., 2005). On the other hand, ΔΨ in the acidophilic archaea, *Picrophilus oshimae* has a negative direction (ca, + 50 to + 100 mV) for ATP production (van de Vossenberg et al., 1998). These facts suggest that a large ΔΨ in alkaliphilic *Bacillaceae* strains plays an important role in alkaline adaptation. The high intensity of H^+^ translocated by the respiratory chain for ATP production under high aeration conditions in *E*. *clarkii* DSM 8720*^T^* may be mainly attributed to ΔΨ. However, the observed ΔΨ (Table 2) alone cannot explain the high ATP productivity in *E*. *clarkii* DSM 8720*^T^*. A large ΔΨ is considered indispensable for energy production in alkaliphilic *Bacillaceae*, and it may be difficult to produce it by intense reaction of the respiratory chain alone. Electron flow in the ordinary respiratory chain is coupled with H^+^ extrusion from the intracellular space to the extracellular space. This H^+^ extrusion across the membrane is hindered by a large ΔΨ, because H^+^ has a positive charge. In addition, the electron flow from cytochrome *c*, which is located on the outer surface of the membrane, to prosthetic groups located on the inner membrane (e.g., cytochrome *a* in cytochrome *c* oxidase) is described above. Furthermore, it is considered that alkaliphilic *Bacillaceae* consume ΔΨ to reduce intracellular pH. Therefore, we propose the use of the Donnan potential (Donnan, 1924; Ohshima and Ohki, 1985; Benarroch and Asally, 2020), which is a congenital factor that takes advantage of the disadvantageous environment that does not consume energy.

### Contribution of ΔΨ to Retain H^+^ at the Outer Surface of the Membrane

H^+^ extrusion in the bulk phase by the respiratory chain in *E*. *clarkii* K24-1U was observed to be very slow (Yoshimune et al., 2010). It appears that the H^+^ extrusion into the bulk phase occurs after H^+^ retention at sites in the vicinity of the outer surface of the membrane. Therefore, H^+^, which is extruded by the respiratory chain, does not appear to be directly extruded into the bulk phase. However, the H^+^, extruded by the respiratory chain the retained on the outer surface of the membrane is released into the bulk phase by the addition of valinomycin or ETH-157, which disrupts ΔΨ (Yoshimune et al., 2010). Therefore, ΔΨ prevents the release of H^+^, which is translocated by the respiratory chain into the bulk phase. The rate of H^+^ translocation in the presence of valinomycin in *E*. *clarkii* K24-1U was approximately 70% that in *B*. *subtilis* under the same experimental conditions. This suggests that the intrinsic H^+^ translocation frequency was reduced by 70% in the alkaliphile as a strategy of reducing H^+^ utilization frequency. Accumulation of H^+^ at the outer surface of the membrane can also be demonstrated by addition of monensin, which is a Na^+^/H^+^ exchange reagent (Yoshimune et al., 2010). Monensin translocates H^+^, previously located to the extracellular surface of the membrane, into the intracellular space, and Na^+^ is located to the intracellular surface of the membrane. The transfer of H^+^, which is extruded by the respiratory chain into the bulk phase is further retarded by the introduction of monensin, which translocates H^+^ located extracellular surface, into intracellular space. These results indicate that ΔΨ influences to the retardation of H^+^ at the outer membrane surface in alkaliphilic *Bacillaceae*.

The much higher per H^+^ efficiency for ATP production in *E*. *clarkii* DSM8720*^T^* than in *B*. *subtilis* IAM 1026 may be attributed to the combination of the H^+^ capacitor effect by cytochrome *c* and high ΔΨ and a high ΔΨ in the vicinity of the outer surface of the membrane. Although the contribution of the H^+^ capacitor effect is unknown, it is difficult to account for the difference in H^+^ efficiency for ATP production between *E*. *clarkii* DSM8720*^T^* and *B*. *subtilis* IAM 1026 based on the difference in the estimated ΔΨ values (Table 2). This may be attributed to either or both the reasons described below. Although ΔΨ was estimated by substitutions of fluorescent changes in the reaction of respiration, it was calibrated by the diffusion potential produced by the different concentrations of K^+^ between intra- and extra-cellular vesicles *plus* valinomycin. One possible reason for this is that the diffusion potential based on valinomycin *plus* K^+^ was not reflected by the real ΔΨ for H^+^ across the membrane. It has been reported that the artificially imposed diffusion potential based on K^+^ has an inferior effect compared to the equal intensity of ΔΨ produced by the respiratory chain in *A*. *pseudofirmus* OF4 (Guffanti and Krulwich, 1994). Another reason is that the estimated ΔΨ is different from the real values in the vicinity of the outer surface of the membrane. It seems reasonable to assume that the force attributed to ΔΨ for H^+^ would be larger for H^+^ located closer to the outer surface of the membrane than H^+^ located in the extracellular bulk phase.

### Basis of Donnan Potential

Negative ion capacity was estimated in the cell extracts containing inside-out membrane vesicles of obligate alkaliphilic *E. clarkii* DSM 8720*^T^* and facultative alkaliphilic *S*. *cohnii* YN-2000 grown at pH 10 and neutralophilic *B*. *subtilis* IAM 1026 grown at pH 7 under high-aeration conditions (Goto et al., 2016). The negative ion capacity of intracellular contents in the two strains of alkaliphiles increased with measured pH between pH 6 and pH 8, whereas that in *B*. *subtilis* IAM 1026 barely changes between pH 6 and pH 10. The negative ion capacity of *E. clarkii* DSM 8720*^T^* and *S*. *cohnii* YN-2000 was 4.7-times and 4.1-times higher than that of *B*. *subtilis* IAM 1026 based on each corresponding intracellular pH. The intracellular materials obtained by two alkaliphiles grown under low-aeration conditions exhibited approximately 72% intensity of negative ion capacity than those under high-aeration conditions at the corresponding intracellular pH (Goto et al., 2016). The intensities of the intracellular negative-ion capacity and the corresponding measured ΔΨ were correlated. Therefore, it is considered that the intracellular negative-ion capacity influences to ΔΨ, which is generated across the membrane. However, it is unclear whether the estimated negative ion capacity includes membrane-permeable ions. The high negative ion capacity in the alkaliphilic strains is probably attributed to the intracellular acidic substances, including proteins, at higher intracellular pH (ca. pH 8–8.2) than in the ordinary neutrophilic strains (pH 6–7). Protein p*I* distribution estimated using the genomes of alkaliphilic, neutralophilic, and acidophilic strains indicates a preference for acidic proteins (p*I* 4.01–5) in alkaliphiles (Lebre and Cowan, 2020).

### Possible Effect of ΔΨ to Membrane Bound Cytochrome *c*-550

Electron transfer-coupled H^+^ transfer in horse heart cytochrome *c* was examined by SAMs cytochrome *c* attached to an Ag electrode produced at different distances connected to the different chain lengths (C_6_–C_16_) of ω-carboxyl alkanethiols (Murgida and Hildebrandt, 2001). Only in the case where the distance between cytochrome *c* and the electrode is short (C_2_), a difference in the electron transfer rate of the H^+^/D^+^ effect is observed: 132 s^–1^ in H_2_O_2_ and 33 s^–1^ in D_2_O. H^+^/D^+^ effect does not occur in cytochrome *c* in the solution. Therefore, these results suggest that the prerequisites for electron transfer coupled with H^+^ transfer are (i) cytochrome *c* forms accumulated, such as a monolayer on the electrode, (ii) accumulated cytochrome probably should be lined up in the correct orientation, and (iii) cytochrome *c* is affected by Coulomb force from the electrode, which may correspond to ΔΨ on the membrane surface. Considering the high ATP productivity in alkaliphilic *Bacillaceae*, it can be assumed that the actual ΔΨ in the vicinity of the outer surface of the membrane is much higher than the estimated values. In addition, as described above, the redox potential of cytochrome *c*-550 immobilized on the gold electrode using SAMs was much lower than that in the redox titration. This suggests that Coulomb force from the electrode induces conformational changes around heme *c*, altering the redox potential (Murgida and Hildebrandt, 2004). Owing to this change in the redox potential of cytochrome *c*, the difference in the redox potential between cytochrome *c*-550 and the direct electron-accepting prosthetic group in cytochrome *c* oxidase (i.e., heme *a*) becomes larger. This means that a very large redox potential gap [+ 7 mV → ca + 250 mV (e.g., heme *a*)] may be necessary to transfer electrons from cytochrome *c*, which is located on the outer surface of the membrane, to the inner prosthetic component (e.g., heme *a*), which is located on the inner membrane under high ΔΨ and consists of strong intracellular negative charges. It is possible that this ΔΨ-dependent redox potential change mechanism regulates electron transfer in the respiratory chain depending on the intensity of the electric field on the outer surface of the membrane of *E*. *clarkii*.

The above-described are alkaliphilic *Bacillaceae* strains that utilize outer-surface-membrane-based ΔΨ consisting of the Donnan potential, which is attributed to the high intracellular pH and the intracellular acidic substances, including proteins. In accordance with the large ΔΨ, the respiratory chain is modified to match the harsh conditions for electron flow from the extracellular side to the intracellular side and translocation of H^+^ from the intracellular side to the extracellular side. The combination of enhanced ΔΨ and specifically modified respiratory components in obligately alkaliphilic *E*. *clarkii*, and influenced the enhancement of respiratory extruded H^+^ protection for ATP production. This system will make a limited amount of H^+^ present in the harsh environment even more effective for utilization of H^+^.

## Conclusion and Perspective

The main sources of bacterial membrane potential consist of charge separations driven by heterogeneous permeable ions across the membrane (V_*G*_) and membrane impermeable substances (V_*D*_) (Benarroch and Asally, 2020). There is a possibility that charge separation driven by heterogeneous permeable ions across the membrane contributes to the total ΔΨ (V_*T*_). Constant investment of energy is necessary to maintain desirable charge separation across the membrane. This might be one of the reasons for the difference in ATP production rate between alkaliphiles and neutralophiles, which is not reflected in difference in growth intensity. Although the maximum ATP synthesis rates in alkaliphiles are approximately 5–13 times higher than that in *B*. *subtilis*, the growth rate of *B*. *subtilis* is higher than those of the alkaliphiles. To understand the breakdown of the overall energy balance of alkaliphilic bacteria, it is necessary to determine the intensity of each constituent factor, V_*G*_, V_*D*_, and the asymmetric surface potential on either side of the membrane produced by the differences in phospholipid head groups.

Based on the molecular features of membrane-bound cytochrome *c* in *E*. *clarkii*, it can be predicted that the segment near the N-terminus produces an H-bond network in the space between the main body cytochrome *c* molecule and the outer surface of the membrane. This configuration may lead to the formation of an H^+^ capacitor on the outer membrane surface. The regulation of horizontal H^+^ transfer on the outer membrane surface is thought to occur in conjunction with the redox reaction of cytochrome *c*. It can be predicted that the order of electron transfer in the tetramer depends on the distance of the molecule from the membrane. Inter-cytochrome *c* molecular horizontal electron transfer may regulate horizontal H^+^ transfer on the outer membrane surface. Cytochromes *c* exhibit very low redox potentials and these potentials will further be decreased depending on the electric field on the outer surface of the membrane. Although the original electron acceptor of cytochrome *c* is cytochrome *c* oxidase, the low redox potentials may include other electron acceptors including NPS or redox centers in complex III. It is believed that a proper H^+^ capacitor function will work with appropriate control of its functions. However, to clarify of the regulations, we must wait for future studies on these mechanisms.

As described above, a high ΔΨ and an H^+^ capacitor produced by the membrane-bound cytochrome *c* are the key points of energy acquisition under high- and low-aeration conditions, respectively (Figure 4). However, the rationales of each strategy in different environments have not yet been understood well. Therefore, we cannot explain the fundamental reasons why highly efficient ATP production can be possible under high aeration conditions although the turnover of the respiratory chain is not high. Since each bioenergetic factor is strongly related to one another (e.g., ΔΨ vs. oxygen consumption rate), the fundamentals in each aeration condition can be determined in further studies.

Alkaliphilic bacteria exhibit a lower respiratory rate than neutralophilic *B*. *subtilis* under high aeration conditions. This could be explained by H^+^ translocation from intracellular space to extracellular space under the presence of high ΔΨ in alkaliphiles. On the contrary, under low aeration conditions, the voltage of the respiratory chain from NADH to O_2_ drops, and H^+^ cannot be translocated in the presence of high ΔΨ. Therefore, alkaliphiles decreased their ΔΨ, and take opt to accumulate H^+^ *via* cytochrome *c*, which possesses a special segment producing an H-bond network on the outer surface of the membrane. It is considered that this accumulated H^+^ exhibits attractive force toward the intracellular side of the membrane in the same way that a normal capacitor that stores electrons exhibits electrostatic attraction.

## Author Contributions

KY and IY designed this study. TG and SO collected the data. IY and TG performed calculations. IY wrote the manuscript. All authors approved the final version.

## Conflict of Interest

The authors declare that the research was conducted in the absence of any commercial or financial relationships that could be construed as a potential conflict of interest.

## Publisher’s Note

All claims expressed in this article are solely those of the authors and do not necessarily represent those of their affiliated organizations, or those of the publisher, the editors and the reviewers. Any product that may be evaluated in this article, or claim that may be made by its manufacturer, is not guaranteed or endorsed by the publisher.
