# Corrigendum: Differences in Bioenergetic Metabolism of Obligately Alkaliphilic *Bacillaceae* Under High pH Depend on the Aeration Conditions

**DOI:** 10.3389/fmicb.2022.903233

**Published:** 2022-04-27

**Authors:** Toshitaka Goto, Shinichi Ogami, Kazuaki Yoshimume, Isao Yumoto

**Affiliations:** ^1^Bioproduction Research Institute, National Institute of Advanced Industrial Science and Technology (AIST), Sapporo, Japan; ^2^Graduate School of Agriculture, Hokkaido University, Sapporo, Japan; ^3^College of Industrial Technology, Nihon University, Narashino, Japan

**Keywords:** bioenergetic mechanism, membrane electrical potential, *Bacillaceae*, *Evansella clarkii*, Donnan effect, H^+^ capacitor, cytochrome *c*, alkaliphilic

In the original article, there was a mistake in the legend for **Figure 2** as published. It described that “The acid residue-abundant segment of Glu^25^-Lys^46^ (Glu^6^-Lys^27^ in the processed protein base) in *S*. *cohnii* DSM 6307^T^ is indicated by green box” at L2 –L3. The correct legend appears below.

**Figure 2**. Amino acid sequence alignment of membrane bound cytochrome *c*-550 from *Evansella clarkii* and other alkaliphilic and neutralophilic *Bacillaceae*. Asn (N)-rich segment, Asn (N)^21^-Asn (N)^43^ [Asn (N)^4^-Asn (N)^26^ in the processed protein base] in the *E*. *clarkii* cytochromes *c* is indicated by red box. The acid residue-abundant segment of Glu^25^-Lys^46^ (Glu^6^-Lys^27^ in the processed protein base) in *S*. *cohnii* DSM 6307^T^ is indicated by red box. N-terminal amino acid residue in processed cytochrome *c*, [Cys (C)] is indicated by blue marker. The blue boxed sequences are heme sequences representing heme-binding site and axical ligands (H and M). Acidic and basic residues are indicated by blue and red letters. Although *A*. *pseudofirmus* OF4 is a facultative alkaliphile, the species *A*. *pseudofirmus* and the presented cytochrome *c* sequence are classified in obligate alkaliphile. Although *Sporosarcina pasteurii* is an obligate alkaliphile, its cytochrome *c* sequence is similar to those in the facultative alkaliphilic strains.

In the original article, there was a mistake in Table 2 as published. The unit for respiratory rate was incorrect. The corrected Table 2 appears below.

**Table 2:** Summary for high aeration and low aeration in obligate alkaliphilic *Evansella clarkii*, facultative alkaliphilic *Sutcliffiella cohnii*, and neutralophilic Bacillussubtilis^*^.

	**Growth rate (μ_max_)^1^**	**Cytochrome *c* content (nmol ·mg^**−1**^)^**1**^**	**ΔΨ (mV)^1^^,^^2^**	**Respiratory rate (μmol ·O_**2**_ min^**−1**^ mg^**−1**^)^**2**^**	**H^**+**^/O^**1**^**	**Maximum ATP synthesis rate^**2**^ (nmol ·min^**−1**^ ·mg^**−1**^)^**2**^**
*E*. *clarkii* DSM8720^T^ Low aeration	0.36	0.89 ± 0.07	−135 ± 8	ND	0.6 ± 0.1 (6)^§^	ND
*E*. *clarkii* DSM 8720^T^ High aeration	0.42	0.36 ± 0.01	−192 ± 3	0.19 ± 0.04	2.2 ± 0.2 (6)	26.2 ± 1.7
*S. cohnii* YN-2000 High aeration	0.38	0.62 ± 0.01	−173 ± 5	0.20 ± 0.03	2.6 ± 0.3 (6)	9.6 ± 0.9
*B*. *subtilis* IAM 1026 Low aeration	0.26	0.18 ± 0.06	−133 ± 13	ND	2.8 ± 0.8 (6)	ND
*B*. *subtilis* IAM 1026 High aeration	0.55	0.21 ± 0.02	−121 ± 7	0.50 ± 0.06	4.9 ± 0.1 (6)	2.0 ± 0.6

* *These data cited from*

1 *Goto (2006) and*

2 *Goto et al. (2016)*.

§ *The numbers in parentheses are based on the theoretically extruded H^+^ in 1/2O_2_ consumption by the respiratory chain*.

The authors apologize for this error and state that this does not change the scientific conclusions of the article in any way. The original article has been updated.

## Publisher's Note

All claims expressed in this article are solely those of the authors and do not necessarily represent those of their affiliated organizations, or those of the publisher, the editors and the reviewers. Any product that may be evaluated in this article, or claim that may be made by its manufacturer, is not guaranteed or endorsed by the publisher.
